# Immunohistochemical identification and assessment of the location of leptin, visfatin and chemerin in the liver of men with different body mass index

**DOI:** 10.1186/s12876-022-02299-6

**Published:** 2022-05-12

**Authors:** I. Kasacka, Ż. Piotrowska, N. Domian, A. Lewandowska, M. Acewicz

**Affiliations:** Department of Histology and Cytophysiology, Medical University of Białystok, Mickiewicza 2C Street, 15-222 Białystok, Poland

**Keywords:** Leptin, Visfatin, Chemerin, Liver, Human, Overweight, Immunohistochemistry, qRT-PCR

## Abstract

**Background:**

Adipokines such as leptin, visfatin and chemerin play a pivotal role not only in the pathogenesis of excessive weight gain but also impact on hepatic metabolism. However, alterations in the production of these peptides in the liver of overweight individuals have not been fully elucidated yet. The aim of the study was to evaluate changes in leptin, visfatin and chemerin biosynthesis in the liver of men with different BMI.

**Methods:**

Fourteen adult men without symptoms from the digestive system were recruited. Research material consisted of liver samples. Study participants were divided into two groups: lean (BMI ≤ 25 kg/m^2^) and overweight subjects (BMI > 25 kg/m^2^). Paraffin liver sections were processed by immunohistochemistry for detection of leptin, visfatin and chemerin. Hepatic expression of leptin, visfatin and chemerin genes was determined by qRT-PCR method.

**Results:**

Increased immunoreactivity for leptin and chemerin, and decreased immunoreaction for visfatin were observed in the liver of overweight men in comparison to lean subjects. Overweight subjects with hepatic steatosis displayed increased immunoreactivity for leptin and weaker immunoreaction against visfatin and chemerin in the liver, compared to individuals with normal organ structure. Expression of leptin and chemerin was enhanced in the liver of overweight individuals, with the highest expression observed in subjects with hepatic steatosis. Conversely, expression of visfatin in the male liver was decreased in overweight subjects and those with and liver steatosis.

**Conclusions:**

The present study proves that the expression of leptin, visfatin and chemerin in the male liver is altered in overweight individuals. Our report also indicates the potential importance of these peptides in hepatic steatosis associated with overweight.

## Background

Leptin, visfatin and chemerin are peptide hormones secreted primarily by adipose tissue and thus classified as adipokines. The presence of these peptides has also been revealed in most other tissues and organs, including the liver, brain, lungs, kidneys, heart, pituitary gland, adrenals, stomach, pancreas, placenta, gonads, skeletal muscle, bone marrow [1–3]. Leptin, visfatin and chemerin have pleiotropic effects and regulate multiple key processes in the body [1–3]. The above-mentioned adipokines are involved in the central regulation of feeding behaviour and energy homeostasis as they affect hunger and satiety centres in the hypothalamus [4–6]. These peptides also influence adipogenesis and lipid accumulation within adipocytes, and hence determine the thickness of adipose tissue [7–9]. Moreover, they modulate insulin secretion and insulin-dependent glucose uptake in target cells, i.e. adipocytes, hepatocytes and muscle cells [10–13].

Leptin is one of the first known peptide hormones associated with excessive weight gain [1, 14, 15]. The list of hormonal factors involved in the development of obesity has been greatly extended in recent years, including visfatin and chemerin [16–21]. Several clinical studies have demonstrated alterations in leptin, visfatin and chemerin biosynthesis in obesity [14–21]. Obese patients display significantly elevated leptin and chemerin levels in blood which correspond with body mass index (BMI), body fat percentage and volume of adipose tissue [14, 16, 17]. Similarly, considerably higher expression of leptin and chemerin genes has been found in adipose tissue of obese individuals [15, 17]. Literature data regarding the role of visfatin in the pathogenesis of obesity is inconsistent. Some authors have shown reduced or unchanged blood levels of visfatin in obese patients while others have demonstrated a positive correlation between circulating peptide levels and the patient’s body mass index (BMI) or visceral fat content [18–21]. In obese individuals, both reduced and increased expression of visfatin in adipose tissue has been noted [20, 21].

Excessive weight gain is associated with an increased risk of fatty liver and the development of nonalcoholic fatty liver disease (NAFLD). Intake of fatty acid that exceeds the liver's ability to metabolise it, leads to intrahepatic accumulation of triglycerides. Steatosis may be further accompanied by hepatitis and fibrosis [22].

Leptin, visfatin and chemerin impact on metabolic activity of hepatocytes. The effect of these peptides on lipid accumulation, lipogenesis, glucose uptake and gluconeogenesis in hepatic cells has been proven [12, 13, 23–28]. In addition, studies on rats have demonstrated that intraperitoneal administration of leptin promoted liver regeneration and hepatocyte proliferation after partial hepatectomy [29]. Other in vitro investigations have shown the protective effect of visfatin on liver cells [30]. Recent clinical studies have revealed abnormal levels of leptin, visfatin and chemerin in the blood and liver of patients with NAFLD, chronic hepatitis and hepatocellular carcinoma [31–42]. These reports also showed that levels of circulating peptides and their expression in the liver correlate with the severity of histopathological changes, i.e. steatosis, fibrosis, inflammation and the degree of liver damage [31–42].

Knowledge regarding changes in the biosynthesis of leptin, visfatin and chemerin in obesity is primarily based on research evaluating theirs levels in the patient’s serum or adipose tissue [14–21]. Far fewer reports concern alterations in the production of these peptides in the human liver under conditions of excessive weight gain [19, 36, 38, 42]. Those studies were conducted mainly on biopsy material collected from severely obese patients, where mRNA for leptin, visfatin and chemerin or peptide levels were measured in liver tissue homogenates [19, 36, 38, 42].

The aim of the study was to immunohistochemically identify and assess leptin, visfatin and chemerin in the liver of lean and overweight males as well as to evaluate hepatic expression of leptin, visfatin and chemerin in men with different BMI.

## Material and methods

### Sample collection

Fourteen adult men without symptoms from digestive system were used in the study. The mean age of study participants was 51.9 ± 2.54 years; body weight was ranged from 67 to 95 kg and patients BMI was ranged from 23.5 kg/m^2^ to 28.7 kg/m^2^. The study participants were divided into two groups: 6 lean subjects with BMI ≤ 25 kg/m^2^ and 8 overweight subjects with BMI > 25 kg/m^2^.

Each study participants showing clinical signs of brain death, was considered to be an organ donor. Irreversible brain damage was confirmed by special clinical examination and angiography (no blood flow within the brain arteries). After diagnosis of brain death and confirmation of death by doctors, samples of liver (about 1 cm^3^) from each body were taken.

Liver samples were immediately fixed in 10% buffered formalin and routinely embedded in paraffin. Sections (4 µm) were stained with hematoxylin–eosin for general histological examination and processed by immunohistochemistry for detection of leptin, visfatin and chemerin.

### Ethical issues

The study protocol was approved by the Ethics Committee at the Medical University of Białystok (R-I-002/345/2007) and written informed consent had previously been obtained from each study participant or from his family member(s).

### Immunohistochemical procedures

Paraffin blocks were cut into 4-µm Sects. (9 liver sections from each subject: 3 section for immunodetection of leptin, 3 section for immunodetection of visfatin and 3 section for immunodetection of chemerin) and attached to positively charged glass slides. In the immunohistochemical study, the EnVision method was used, as previously described by Kasacka et al. [43]. Immunohistochemistry was performed, using an REAL™ EnVision™ Detection System, Peroxidase/DAB, Rabbit/Mouse detection kit (K5007; Dako, Glostrup, Denmark). Immunostaining was performed by the following protocol: paraffin-embedded sections were deparaffinized and hydrated in pure alcohols. For antigen retrieval, the sections were subjected to pretreatment in a pressure chamber heated for 1 min at 21 psi (one pound force per square inch (1 psi) equates to 6.895 kPa, the conversion factor has been provided by the United Kingdom National Physical Laboratory) at 125 °C, using Target Retrieval Solution S 1699 for leptin and visfatin (Dako, Glostrup, Denmark) and Target Retrieval Solution with pH of 9.0 S 2367 for chemerin. After cooling down to room temperature, the sections were incubated with DAKO Peroxidase Block S 2023 (Dako, Glostrup, Denmark) for 5 min to block endogenous peroxidase activity. Subsequently sections were incubated with the primary antibody to leptin (Rabbit polyclonal antibody to leptin H-003-12 Phoenix Pharmaceutical Inc., USA), primary antibody for visfatin (Rabbit polyclonal antibody to visfatin H-003-93 Phoenix Pharmaceutical Inc., USA) and chemerin (Mouse polyclonal antibody to chemerin ab72965, Abcam). Primary antibodies were previously diluted in Antibody Diluent Background Reducing (S 3022 Dako, Glostrup, Denmark) in relation to 1: 10,000 for leptin, 1:15,000 for visfatin and 1:250 for chemerin. Sections with leptin and visfatin antibody were incubated overnight at 4 °C, section with chemerin antibody were incubated for 90 min at room temperature (incubation performed in a humidified chamber). The procedure was followed by incubation with secondary antibody (conjugated to horseradish peroxidase-labelled polymer) (60 min for visfatin and leptin, 30 min for chemerin). The bound antibodies were visualized by 1-min incubation with liquid 3,3′-diaminobenzidine substrate chromogen. The sections were finally counterstained in hematoxylin QS (H - 3404, Vector Laboratories; Burlingame, CA), mounted, covered and evaluated under a light microscope. Appropriate washing with Wash Buffer (S 3006 Dako, Glostrup, Denmark) was performed between each step.

Specificity tests performed for leptin, visfatin and chemerin antibodies included a negative and positive control. In the negative control antibodies were replaced by normal rabbit serum (Vector Laboratories; Burlingame, CA) with appropriate dilution. Omission of the primary antibody in immunohistochemical reactions was negative. Positive controls were performed according to the manufacturer's instructions on human adipose tissue for leptin and on human liver for visfatin and chemerin.

Histological preparations were evaluated using an Olympus BX43 light microscope (Olympus 114 Corp., Tokyo, Japan) with an Olympus DP12 digital camera (Olympus 114 Corp., Tokyo, Japan) and documented.

### Quantitative analysis

Immunohistochemical reactions were carried out on three sections for each antibody. Five randomly selected microscopic fields (each field 0.785 mm^2^, 200 × magnification (20 × lens and 10 × eyepiece)) from each liver section were documented using an Olympus DP12 microscope camera. Each digital image of the liver section was morphometric evaluated using NIS Elements AR 3.10 Nikon for microscopic image analysis.

The intensity of the immunohistochemical reaction for leptin, visfatin and chemerin was measured in each image and determined using a gray scale level of 0–255, where the value of the completely white or light pixel is 0, while the completely black pixel is 255.

### Real-time PCR

Total RNA was isolated using the Total RNA isolation from FFPE Samples (Macherey–Nagel). Quantification and quality control of total RNA was determined using the spectrophotometer - NanoDrop 2000 (ThermoScientific, Waltham, MA, USA). Only RNA samples for which the absorbance ratio at wavelength 260 nm/280 nm was 1.8–2.1 were adopted for the next analysis steps. The mentioned absorbance ratio proves that isolated RNA is of high quality. An aliquot of 1 µg of total RNA was reverse transcribed into cDNA using iScript™ Advanced cDNA Synthesis Kit for RT-qPCR (BIO-RAD, Berkeley, California, USA). Synthesis of cDNA was performed in a final volume of 20 μl using an Thermal Cycler (Model SureCycler 8800, Aligent Technologies). For reverse transcription, the mixtures were incubated at 46 °C for 20 min then heated to 95 °C for 1 min and finally rapidly cooled at 4 °C.

Quantitative real-time PCR (qRT-PCR) reactions were performed using Stratagene Mx3005P (Aligent Technologies) with the SsoAdvanced™ Universal SYBER® Green Supermix (BIO-RAD, Berkeley, California, USA). Specific primers for the leptin (*LEP*), visfatin (*NAMPT*), chemerin (*RARRES2*), and *GAPDH* were designed by BIO-RAD Company. The housekeeping gene *GAPDH* was used as the reference gene for quantification. In order to determine the amounts of tested genes expression levels, standard curves for each gene separately were constructed with serially diluted PCR products. PCR products were obtained by amplification of cDNA using specific primers as follows *LEP* (qHsaCID0017538, BIO-RAD), *NAMPT* (qHsaCED0043104, BIO-RAD), *RARRES2* (qHsaCID0017608, BIO-RAD) and *GAPDH* (qHsaCED0038674, BIO-RAD). qRT-PCR was carried out in a dublete in a final volume of 10 μl under the following conditions: 2 min polymerase activation at 95 °C, 5 s denaturation at 95 °C, 30 s annealing at 60 °C for 40 cycles. PCR reactions were checked by including no-RT-controls, by omission of templates, and by melting curve to ensure only a single product was amplified. The relative quantification of gene expression was determined by comparison of values of Ct using the ΔΔCt method. All results were normalized to *GAPDH*.

### Statistical analysis

All data were analysed for statistical significance using software computer package Statistica Version 12.0. The mean values were computed automatically; significant differences were determined by one-way ANOVA test; p < 0.05 was accepted as significant**.**

## Results

Clinical basal characteristics of the subjects, including mean values of age, body weight, height and body mass index (BMI), are presented in Table 1. Study participants belonged to the middle age group (around 50 years old). Individuals were divided into two groups: men with normal body weight (BMI ≤ 25 kg/m^2^) and overweight subjects (BMI > 25 kg/m^2^).

**Table 1:** Average age [years], body weight [kg], height [cm] and BMI [kg/m^2^] of men (mean ± SE)

Group of men	Age [years]	Weight [kg]	Height [cm]	BMI [kg/m^2^]
Control	47.3 ± 3.62	79.2 ± 2.63	180.3 ± 2.55	24.3 ± 0.24
Overweight	55.2 ± 3.18	87.2 ± 1.85*	178.9 ± 0.91	27.2 ± 0.36*

*p < 0.05 overweight vs control group

**Fig. 1:**
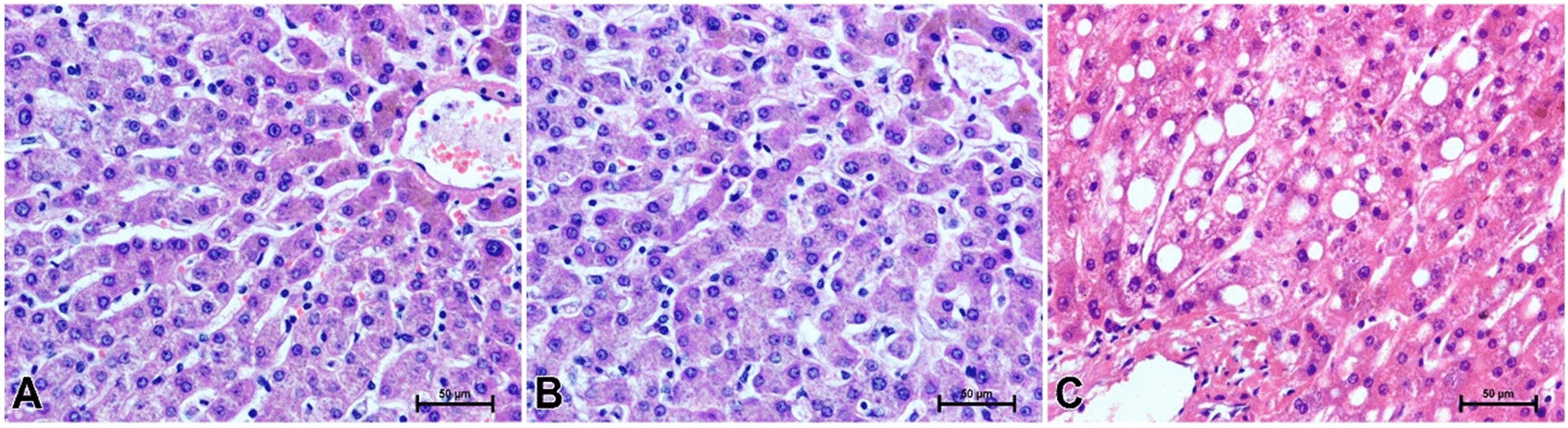
Routine H & E staining of men liver. **A** Man with normal body weight, **B** overweight man without noticeable changes in liver structure, **C** liver steatosis in some of overweight man

Routine histopathological examination did not reveal any changes in liver morphology in men with normal body weight (Fig. 1A). In the group of overweight men, some subjects had normal liver structure (Fig. 1B) while others had significant histopathological changes in the organ (Fig. 1C). Vacuolar or fatty degeneration of hepatocytes was observed in the livers of some overweight males (Fig. 1C).

**Fig. 2:**
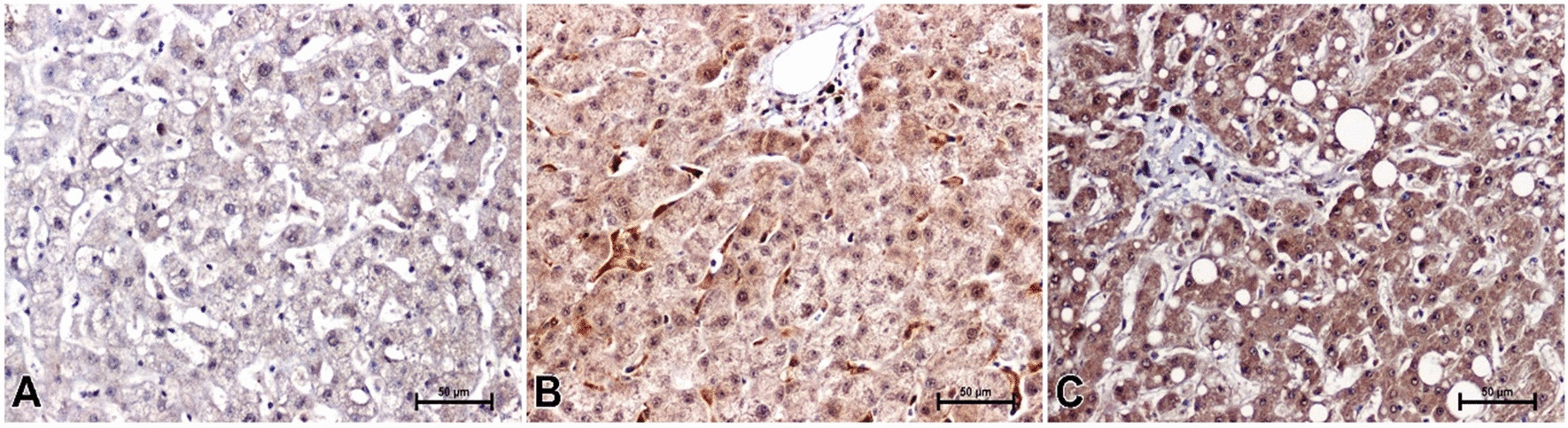
Result of leptin-immunostaining in liver of men. **A** Man with normal body weight, **B** overweight man without noticeable changes in liver structure, **C** overweight man with liver steatosis

A positive immunohistochemical reaction for leptin was noted in the liver of all studied men (Fig. 2A–C). In individuals with normal body weight, intensity of leptin immunoreaction in the liver was moderate (Fig. 2A). Immunoreactivity for leptin in the liver of overweigh subjects was higher when compared to lean participants (Fig. 2B). The strongest immunoreactivity for leptin was noted in the liver of overweight males with hepatic steatosis, compared to subjects with normal hepatic structure and those with a lower BMI (Fig. 2C).

**Fig. 3:**
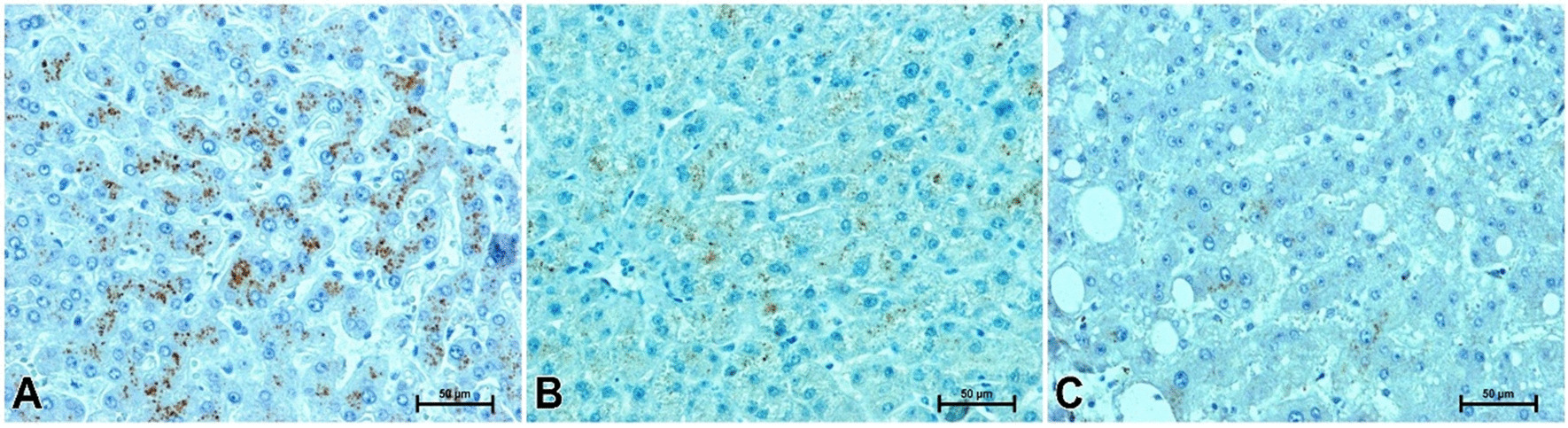
Immunoidentification of visfatin in liver of men. **A** Man with normal body weight, **B** overweight man without noticeable changes in liver structure, **C** overweight man with liver steatosis

The immunohistochemical identification of visfatin gave a positive reaction in the liver of all studied men, except for single cases of overweight subjects with liver steatosis. Immunolocation of visfatin revealed its presence in hepatocytes in the form of small brown-coloured granules dispersed in the cell cytoplasm (Fig. 3A–C). Strong visfatin immunoreactivity was noted in the liver of men with normal body weight (Fig. 3A). In the liver of men with a higher BMI, immunoreactivity for visfatin was far weaker. Positive reaction results were observed in a smaller number of hepatocytes (Fig. 3B, C). In overweight subjects with normal liver structure, visfatin immunostaining was weak to moderate (Fig. 3B) while, in hepatic steatosis, immunoreactivity of visfatin was minimal or negative (Fig. 3C).

**Fig. 4:**
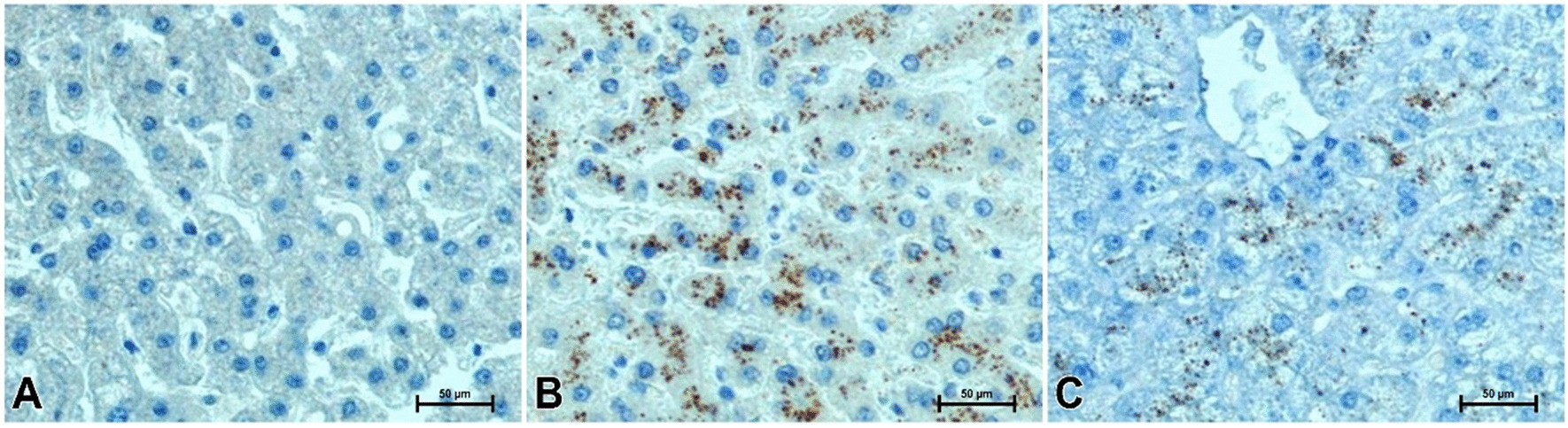
Immunohistochemical reaction determining chemerin in liver of men. **A** Man with normal body weight, **B** overweight man without noticeable changes in liver structure, **C** overweight man with liver steatosis

Immunodetection of chemerin gave a very weak or almost undetectable reaction in the liver of men with normal body weight (Fig. 4A). Far higher intensity of reaction showing chemerin was found in the liver of overweight subjects compared to those with a lower BMI (Fig. 4B, C). In the cytoplasm of liver cells in overweight men without noticeable changes in the structure of the organ, numerous brown-stained chemerin-immunopositive granules were observed (Fig. 4B). In overweight males with histopathological changes in the liver, the reaction result showing chemerin was significantly weaker compared to that observed in the previous group. This applies both to the intensity of reaction and the number of hepatocytes containing chemerin granules (Fig. 4C).

Table 2 shows the results of measurements of the intensity of immunostaining using computer image analysis. In the liver of overweight men higher leptin and chemerin immunoreaction but lower visfatin immunoreactivity were found compared to subjects with normal body weight. Overweight individuals with hepatic steatosis displayed stronger leptin immunoreactivity in the liver compared to overweight men without histopathological changes in the organ, whereas the intensity of reaction against visfatin and chemerin was weaker in overweight men with fatty liver compared to overweight subjects with normal hepatic structure.

**Table 2:** The intensity of immunoreaction determining leptin, visfatin and chemerin in liver of men (mean ± SE)

Group of men		Intensity of immunohistochemical reaction in man liver Scale from 0 (white pixel) to 255 (black pixel)		
		Leptin	Visfatin	Chemerin
Control	*Overall*	77.3 ± 1.77	169.8 ± 2.67	60.2 ± 1.21
Overweight	*Overall*	134.6 ± 2.62*↑	109.4 ± 2.22*↓	121.2 ± 1.82*↑
	Normal hepatic structure	111.6 ± 3.09*↑	125.8 ± 2.70*↓	131.0 ± 2.08*↑
	Hepatic steatosis	152.5 ± 2.79*↑ ^♯^↑	91.0 ± 2.44*↓ ^♯^↓	112.3 ± 2.65*↑ ^♯^↓

*p < 0.05 overweight vs control group

^♯^p < 0.05 overweight with hepatic steatosis vs overweight with normal hepatic structure

↓Weakening of immunohistochemical reaction

↑Intensification of immunohistochemical reaction

The qRT-PCR analysis revealed significant changes in the expression of genes coding leptin, visfatin and chemerin in the liver of overweight males with histopathological changes in the organ. Expression of the leptin and chemerin genes was enhanced while expression of the visfatin gene was decreased in the liver of overweight men in comparison to subjects with a lower BMI. Overweight males with hepatic steatosis showed the highest expression of leptin and chemerin but lowest expression of visfatin compared to overweight men with normal liver structure or to lean individuals (Table 3).

**Table 3:** Expression of genes coding leptin (*LEP*), visfatin (*NAMPT*) and chemerin (*RARRES2*) in liver of men (mean ± SE)

Group of men		Expression of genes (mean ± SE)		
		*LEP* (leptin)	*NAMPT* (visfatin)	*RARRES2* (chemerin)
Control	*Overall*	0.21 ± 0.008	1.85 ± 0.086	1.36 ± 0.121
Overweight	*Overall*	1.33 ± 0.353*↑	0.34 ± 0.086*↓	8.31 ± 1.111*↑
	Normal hepatic structure	0.58 ± 0.083*↑	0.53 ± 0.035*↓	6.23 ± 0.693*↑
	Hepatic steatosis	2.08 ± 0.232*↑ ^♯^↑	0.15 ± 0.006*↓ ^♯^↓	10.40 ± 1.160*↑ ^♯^↑

*p < 0.05 overweight vs control group

^♯^p < 0.05 overweight with hepatic steatosis vs overweight with normal hepatic structure

↓Reduced expression

↑Intensified expression

## Discussion

Overweight and obesity are leading risk factors of liver steatosis and NAFLD [22]. Considering that adipokines such as leptin, visfatin and chemerin play a pivotal role not only in regulating energy balance and body weight but also influence hepatic metabolism, it appears worth to evaluate the effect of excessive weight gain on the levels of these adipokines in the human liver [4–9, 12, 13, 23–28]. The current state of knowledge regarding hepatic leptin, visfatin and chemerin biosynthesis in obese patients is not comprehensive. There are only few reports in the available literature investigating expression of these adipokines in the liver of patients with severe obesity while in patients with overweight or mild obesity their hepatic production has not been examined [19, 36, 38, 42].

The aim of the current study was immunohistochemical detection and assessment of leptin, visfatin and chemerin in the liver of men with different BMI as well as evaluation of leptin, visfatin and chemerin hepatic expression in lean and overweight males.

Our study revealed increased immunoreactivity for leptin and chemerin but reduced immunoreaction for visfatin in the liver of overweight participants compared to subjects with normal body weight. Overweight individuals with hepatic steatosis displayed increased immunoreactivity for leptin, and lower intensity of immunoreaction against visfatin and chemerin in the liver compared to subjects without histopathological changes in the organ. Expression of the leptin and chemerin genes was enhanced in the liver of overweight individuals compared to those with normal body weight, with the highest expression observed in overweight subject with hepatic steatosis. Expression of the visfatin gene was decreased in the liver of overweight men in comparison to lean subjects, with the lowest expression noted in overweight men with liver histopathological changes in the liver.

The findings of the present study are in line with a report by Kukla et al. [36] and Kajor et al. [38] who observed a decrease in visfatin expression and an increase in chemerin expression in the liver concomitant with increasing body weight. Our results are also consistent with those published by Dahl et al. [30], Kajor et al. [38] and Krautbauer et al. [41] who demonstrated reduced visfatin and enhanced chemerin expression in the liver of individuals with hepatic steatosis and NASH compared to subjects with normal liver structure. The findings of the present study are also in agreement with those of Chitturi et al. [31] who showed elevated leptin levels in the blood of individuals with NASH and demonstrated a direct correlation between peptide serum levels and severity of hepatic steatosis. In contrast to our findings, Auguet et al. [19] observed higher visfatin expression in the liver of morbidly obese women in comparison to lean subjects. In a study on severely obese people, Moschen et al. [42] noticed that weight loss after bariatric surgery was accompanied by a decrease in visfatin mRNA and visfatin–immunoreactivity in the patients' liver. At the same time, the authors did not observe any significant changes in leptin expression or peptide content in the liver following a reduction in body weight. Discrepancies in the results of the above-mentioned reports may be explained by various degrees of obesity in studied individuals. In the present study, the BMI of examined men did not exceed 30 kg/m^2^ while in investigations by Auguet et al. [19] and Moschen et al. [42] the BMI of study participants was above 40 kg/m^2^.

Calorie intake in the form of fatty compounds impacts on the biosynthesis of leptin, visfatin and chemerin in the liver. Krautbauer et al. [41] and Sheng et al. [44] demonstrated that rodents on a high fat diet (HFD) had enhanced expression of leptin and chemerin in liver compared to those receiving regular chow. In a study on healthy women, Chamberland et al. [45] observed a significant decrease in blood levels of chemerin and leptin after a 72-h fast. Other researchers have revealed a reduction in visfatin mRNA levels in the liver of mice subjected to HFD compared to those fed regular chow [26]. Tauriainen et al. [46] demonstrated that caloric restriction enhanced visfatin expression in the liver of mice. Given the above, alterations in leptin, visfatin and chemerin immunoreactivity as well as their expression in the liver of overweight males observed in the present study might be related to a higher intake of calories or lipids by those subjects in comparison to lean individuals.

Leptin, visfatin and chemerin are involved in pathological processes leading to hepatic complications. Recent clinical investigations have indicated abnormal levels of these peptides in the blood and liver of patients with NAFLD, chronic hepatitis and hepatocellular carcinoma [31–42]. In subjects with liver disease, blood and hepatic levels of the studied peptides were significantly correlated with the degree of organ steatosis, fibrosis and inflammation [31–42].

The above-mentioned adipokines affect lipid metabolism and deposition in the liver. It was demonstrated that intravenous injection of leptin significantly reduced triglyceride concentration in the rat liver [23]. Huynh et al. [24] revealed that mice with ablated leptin signalling have increased hepatic lipid accumulation. Tao et al. [27] demonstrated that overexpression of the visfatin gene leads to a decrease in triglyceride levels in hepatic cells, whereas its inactivation results in an increase in lipid content in hepatocytes. Wang et al. [26] showed that treatment with an visfatin inhibitor aggravated hepatic steatosis in HFD-fed mice. In this manner, changes in leptin and visfatin expression and peptide content in the liver of subjects with a higher BMI observed in the current study might constitute a possible mechanism participating in fatty degeneration of the liver in overweight and obesity.

In a proportion of individuals, NAFLD occurs with further organ inflammation and fibrosis, progressing to non-alcoholic steatohepatitis (NASH) [22]. Experimental data have revealed that leptin and visfatin potentiate collagen production by stellate cells and promote fibrotic changes in the liver [47, 48]. Among multiple important biological actions, adipokines also have immunomodulatory properties. Leptin, visfatin and chemerin act as proinflammatory agents and chemotactic factors for immune cells, and thus might be involved in the process of hepatitis [47, 49]. In view of the above, altered leptin, visfatin and chemerin expression in the liver of overweight men observed in the present study might suggest participation of these adipokines in the development of histopathological changes in the liver in the course of excessive weight gain.

Obesity is increasingly regarded as a condition associated with chronic systemic low-grade inflammation. Elevated levels of inflammatory markers, e.g. chemokines, interleukins (IL), tumor necrosis factors (TNF) have been observed in the blood of overweight and obese patients [50]. Available literature indicates the stimulating effect of cytokines (e.g. IL-1, IL-6, TNF-α) on leptin and chemerin biosynthesis in adipose tissue [51, 52]. On the other hand, IL-6 and TNF-α negatively regulate visfatin expression in adipocytes [53, 54]. It is possible that changes in leptin, visfatin and chemerin expression in the liver of overweight men observed in the present study may be associated with general or local intensification of inflammatory processes.

Overweight and obese individuals are at increased risk of developing insulin resistance and consequently diabetes [55]. Leptin, visfatin and chemerin play a significant role in controlling glucose homeostasis in the body. These adipokines modulate pancreatic insulin production and insulin-dependent glucose uptake by adipocytes, hepatocytes and skeletal muscles. They also affect gluconeogenesis, glycogenolysis and glucose release in hepatic cells [10–13, 25]. Conversely, the expression of leptin, visfatin and chemerin might be modulated by insulin and glucose. Luque-Ramírez et al. [56] noted increased circulating leptin and chemerin levels in subjects administered a 75 g glucose solution. Several experimental data prove that glucose as well as insulin potentiate the expression of leptin and chemerin, but inhibit the expression of visfatin in adipocytes and hepatocytes [30, 57–59]. Considering the above, altered biosynthesis of leptin, visfatin and chemerin in the liver of overweight individuals reported in the current study might result from insulin resistance and disturbed glucose management in hepatic cells or hyperinsulinemia and hyperglycemia which accompany obesity.

The present study undoubtedly proves that the expression of leptin, visfatin and chemerin in the human liver is altered in overweight and obesity. Recent literature data provide increasing evidence that adipokines play a key role in the pathogenesis and progression of NAFLD, which is common in overweight people. Obesity itself is associated with so-called systemic inflammation, which may possibly be involved in the pathogenesis of NAFLD. Our research has significant clinical importance mainly due to the increase in the incidence of NAFLD and the related increase in morbidity and mortality [36, 38].

Recent studies prove that leptin is responsible not only for the development but also the progression of NAFLD. Elevated leptin levels are associated with the severity of the disease. Leptin is the only adipokine that has been defined as an "adipokine drug" and has been approved by the US Food and Drug Administration. Treatment with recombinant human leptin-metreleptin is currently underway. Results from intervention studies assessing the efficacy of metreleptin in NAFLD related to lipodystrophy are also awaited [60].

Chemerin contributes to inflammatory processes because it is associated with visceral adipose tissue macrophages, hepatic CD68 cell expression, and proinflammatory cytokines such as hepatic TNF-α expression.

Due to the important role of adipokines in the pathogenesis of NAFLD, it can be assumed that their level modification has a positive effect on liver histology. Interestingly, numerous pharmacological agents used to treat NAFLD modulate adipokine levels. An example of a substance related to the level of adipokines such as adiponectin and chemerin is the widely used metformin, which has a hepatoprotective effect [61].

Literature data indicate that visfatin may be involved in the development of NAFLD through: regulation of hepatic inflammation, glucose homeostasis and insulin resistance. Therefore visfatin is used in predictive models to distinguish NASH from simple steatosis. It is worth mentioning the positive influence of probiotics on NAFLD. An example is the “Symbiter” probiotic, which reduces liver fat, TNF-α and IL-6 levels and the activity of transaminases in NAFLD patients, and the modulation of the intestinal microflora may be a breakthrough in the treatment of this disease entity [62].

Despite the extensive research that has been carried out so far, a significant number of issues remain controversial and further detailed research is needed in this direction. New evidence could lead to a better understanding of the pathogenetic NAFLD as well as provide new non-invasive diagnostic and therapeutic approaches.

## Data Availability

Datasets used and/or analyzed in the study are available from the corresponding author on request.
